# Heterochromatin Protein 1 (HP1) Proteins Do Not Drive Pericentromeric Cohesin Enrichment in Human Cells

**DOI:** 10.1371/journal.pone.0005118

**Published:** 2009-04-08

**Authors:** Ángel Serrano, Miriam Rodríguez-Corsino, Ana Losada

**Affiliations:** Chromosome Dynamics Group, Molecular Oncology Programme, Spanish National Cancer Research Centre (CNIO), Madrid, Spain; University of Munich and Center of Integrated Protein Science, Germany

## Abstract

Sister chromatid cohesion mediated by cohesin is essential for accurate chromosome segregation. Classical studies suggest that heterochromatin promotes cohesion, but whether this happens through regulation of cohesin remains to be determined. Heterochromatin protein 1 (HP1) is a major component of heterochromatin. In fission yeast, the HP1 homologue Swi6 interacts with cohesin and is required for proper targeting and/or stabilization of cohesin at the centromeric region. To test whether this pathway is conserved in human cells, we have examined the behavior of cohesin in cells in which the levels of HP1 alpha, beta or gamma (the three HP1 proteins present in mammalian organisms) have been reduced by siRNA. We have also studied the consequences of treating human cells with drugs that change the histone modification profile of heterochromatin and thereby affect HP1 localization. Our results show no evidence for a requirement of HP1 proteins for either loading of bulk cohesin onto chromatin in interphase or retention of cohesin at pericentric heterochromatin in mitosis. However, depletion of HP1gamma leads to defects in mitotic progression.

## Introduction

Sister chromatid cohesion is one important mechanism for the cell to ensure faithful chromosome segregation. A physical linkage between the sister chromatids is established by a multiprotein complex named cohesin at the time of DNA replication and persists until all chromosomes are properly aligned on the metaphase spindle. Cohesin is a ring-shaped complex that consists of a heterodimer of Structural Maintenance of Chromosomes (SMC) subunits, SMC1 and SMC3, the kleisin subunit Scc1 (also known as Mcd1/Rad21), and Scc3/SA [1]–[3]. Cohesin is loaded on chromatin in early G1 in vertebrate cells and establishes cohesion during S phase [4], [5]. At the onset of mitosis, most cohesin dissociates from chromatin by the so-called prophase pathway that involves Aurora B and Polo kinases, as well as a number of additional cohesin-interacting factors [6]–[11]. A small population of cohesin, enriched at the centromeric region, remains on chromatin until the onset of anaphase. At this time complete dissolution of cohesion occurs by cleavage of Scc1 by separase [12], [13].

Mapping of cohesin binding sites by chromatin immunoprecipitation analysis in yeast shows a clear enrichment of the complex around centromeres [14]–[16]. Similar studies of cohesin distribution in metazoa have been reported recently, but they exclude the repeated sequences of heterochromatin in which centromeres are located [17]–[19]. Nevertheless, immunofluorescent staining of mitotic chromosomes from Drosophila, Xenopus and human cells evidences the accumulation of cohesin in the centromeric region [12], [20]–[22]. This accumulation likely responds to the need to resist the spindle microtubule pulling forces in mitosis and meiosis [23]. It is unknown whether cohesin enrichment is the result of increased recruitment of cohesin around centromeres in interphase, of preferential dissociation of cohesin from chromosome arms in early mitosis, or both. A family of proteins known as “shugoshins” do indeed protect centromeric cohesin from dissociation in prophase [24].

Metazoan centromeres are embedded in heterochromatin. Classically, this chromatin domain is defined as the portion of the genome that retains deep staining with DNA-specific dyes and remains cytologically condensed throughout the cell cycle. It is mainly composed of repetitive sequences -satellite DNAs and transposons- and contains few genes [25]. Nucleosomes of heterochromatin regions are usually hypoacetylated and histone H3 is methylated in Lysine 9 (H3K9Me) [26]. Another prominent “mark” of heterocromatin is the presence of Heterochromatin Protein 1 (HP1). Initially identified in *Drosophila melanogaster*, HP1 has homologues in various organisms, from *Schizosaccharomyces pombe* (Swi6) to mammals, in which three HP1 isoforms (alpha, beta and gamma) have been identified [27], [28]. Although HP1 proteins are primarily associated with pericentric heterochromatin, they have also been mapped to euchromatic sites as well as telomeres [29]–[31]. The HP1 isoforms share a common structural organization with a chromodomain and a chromoshadow domain in the amino- and carboxy-terminus, respectively, separated by a flexible hinge region. The chromodomain is responsible for binding to H3K9Me, although additional factors are required to specify HP1 targeting [32]–[34]. The chromoshadow domain mediates interactions with a number of nuclear proteins that include the large subunit of the chromatin assembly factor-1 (CAF-1), the histone H3 lysine metyltransferase Suv39h or the DNA methyl transferases Dnmt1 and Dnmt3a [35].

A number of classical observations suggest a role of heterochromatin in sister chromatid cohesion. For example, the arms of Drosophila Y chromosome, composed mainly of heterochromatin, maintain their close apposition when cells are arrested in mitosis with colchicine [36]. In mammalian cells, the order in which chromosomes separate in anaphase correlates with their amount of pericentric heterochromatin, probably because they require more time to completely dissolve cohesion [37]. The question is whether heterochromatin “stickiness” is due to cohesin-mediated cohesion and how it is regulated. In *S. pombe*, mutants in the HP1 homologue Swi6 lack cohesin in the outer centromeric repeat region and, as a consequence, show chromosome segregation defects [38], [39]. Supporting the conservation of this mechanism in higher eukaryotes, mouse cells deficient for enzymes responsible for H3K9 tri-methylation (Suv39h1 and Suv39h2), in which there is no apparent enrichment of HP1 in pericentric heterochromatin, showed reduced cohesion in the pericentric major satellite [40], [41]. However, cohesin appears to be present at this region in Suv39h^−/−^ cells [42]. In Drosophila mutants for the H3K9 methyltransferase there is a slight reduction in the amount of cohesin in the 1.688 pericentric satellite [43]. Nevertheless, larvae expressing reduced or mutant versions of HP1 show no apparent defects in pericentromeric cohesion [44].

To further explore the function of heterochromatin in cohesin regulation in human cells, we chose to alter its composition by depleting HP1 proteins by means of RNA interference. After a 90% reduction in the levels of the HP1 isoforms, we found no effect on the binding of bulk cohesin to chromatin in interphase cells and no effect on the pericentric accumulation of cohesin in metaphase chromosomes. Treatments with a histone deacetylase inhibitor or a DNA methylation inhibitor that reduce the binding of HP1 to heterochromatin have also no consequences on cohesin behavior. We conclude that, unlike fission yeast, human HP1 proteins are not responsible for the enrichment of cohesin around centromeres.

## Results

### Localization of HP1 proteins in HeLa cells

**Figure 1:**
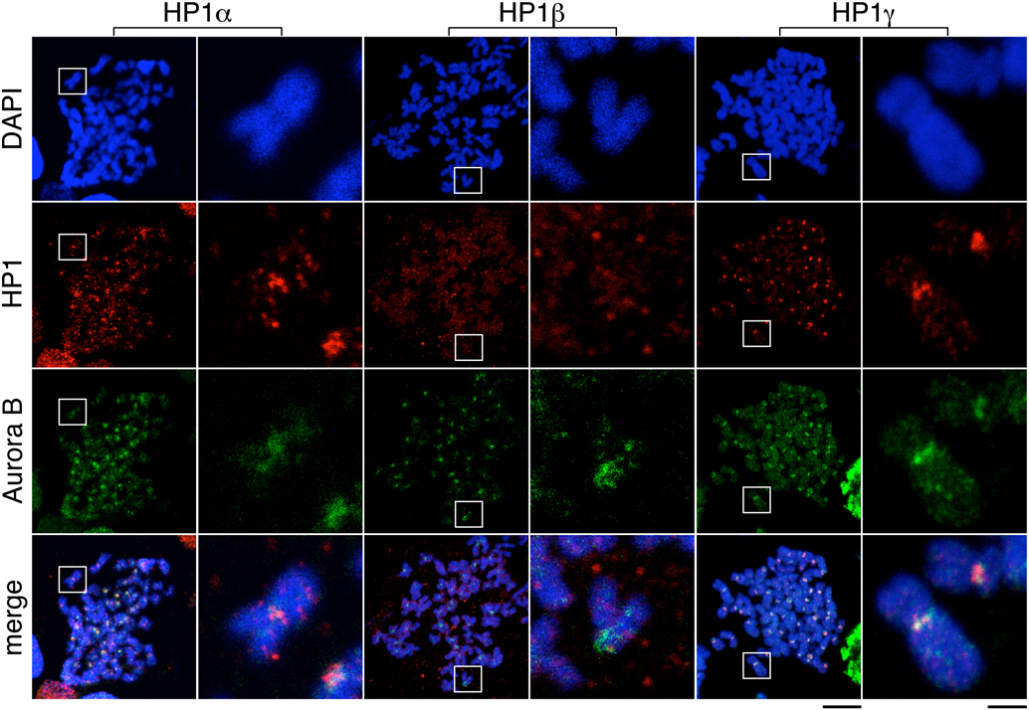
Centromeric localization of HP1 proteins on mitotic chromosomes. Metaphase chromosome spreads of HeLa cells were stained with antibodies against HP1alpha, HP1beta or HP1gamma (red), anti-Aurora B (green) and DAPI (blue). A whole metaphase is shown on the left (scale bar, 10 micrometers) and a single representative chromosome is shown on the right (scale bar, 2 micrometers).

Immunofluorescent staining of HeLa cells with antibodies that specifically recognize each one of the three HP1 isoforms indicates that they are all nuclear proteins that are bound to chromatin and accumulate on foci in interphase (Figure S1, top). Most HP1 dissociates from chromatin in mitosis (Figure S1, bottom). The mitotic populations of HP1alpha and HP1gamma can be detected at the pericentric region of condensed chromosomes, where they co-localize with Aurora B (Figure 1). We failed to detect a similar staining pattern for HP1beta. Cohesin is also present in the inner centromeric region of mitotic chromosomes, although it is very difficult to detect (see below).

### No evidence of physical interaction between cohesin and HP1 isoforms in human cells

**Figure 2:**
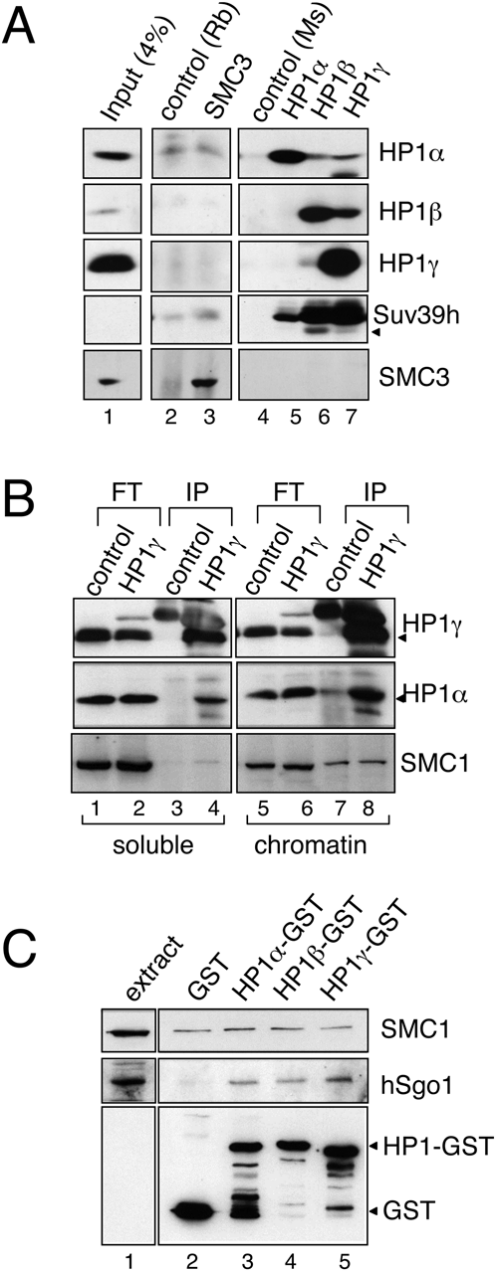
No physical interaction between HP1 proteins and cohesin. (A) Immunoprecipitation of native proteins from HeLa nuclear extracts with non-immune rabbit IgG (lane 2) or mouse preimmune serum (lane 4) and specific antibodies against cohesin SMC3 subunit (lane 3), HP1alpha (lane 5), HP1beta (lane 6) and HP1gamma (lane 7). The immunoprecipitates were analyzed by immunoblotting with the indicated antibodies. An aliquot of the extract was also loaded (lane 1). (B) Immunoprecipitation reactions (IP) carried out with non-immune mouse IgG (control) or anti-HP1gamma from the soluble (lanes 3–4) and chromatin-bound fractions (lanes 7–8) of HeLa cells treated with 1% paraformaldehyde were analyzed by immunoblotting. Aliquots of the corresponding flowthroughs (FT, lanes 1–2 and 5–6) were also loaded. (C) Aliquots of a HeLa cell nuclear extract were incubated with GST agarose bound to GST, HP1alpha-GST, HP1beta-GST or HP1gamma-GST fusion proteins (lanes 2–5, respectively). After washing, the agarose-bound proteins were analyzed by immunoblotting. An aliquot of the HeLa cell extract was also analyzed (lane 1).

Co-immunoprecipitation of Swi6/HP1 and the cohesin subunit Psc3 (Scc3/SA) was detected in fission yeast cell extracts after cross-linking [39]. We first used total cell and nuclear extracts to carry out immunoprecipitation reactions with antibodies against both cohesin and the HP1 isoforms (Figure 2A and data not shown). We did not find cohesin in the HP1 immunoprecipitates and *vice versa*, although we could detect interactions between the HP1 isoforms as well as a small amount of the Suv39h methyltransferase. We next tested the immunoprecipitation of HP1gamma after cross-linking with 1% formaldehyde, a condition similar to the one used in *S. pombe* cells. HP1gamma could be immunoprecipitated efficiently from both the soluble and the chromatin-bound fractions, but no cohesin was pulled-down along with HP1gamma. HP1alpha was again found in the HP1gamma immunoprecipitates (Figure 2B). As an alternative approach, GST-tagged versions of the HP1 proteins were purified from bacteria on glutathione agarose beads and incubated with a HeLa cell nuclear extract. The small amount of cohesin detected likely reflects non-specific binding since it was also detected with GST alone (Figure 2C, lane 2). In contrast, hSgo1 was found to interact specifically with all three HP1 isoforms under this condition, in agreement with a recent report [45]. When the GST-tagged HP1 proteins were incubated with cohesin complexes immunoprecipitated from human cell extracts, no specific interaction could be observed either (data not shown). Thus, it is unlikely that mammalian cohesin interacts directly with HP1 proteins.

### Knock down of HP1 isoforms by siRNA

**Figure 3:**
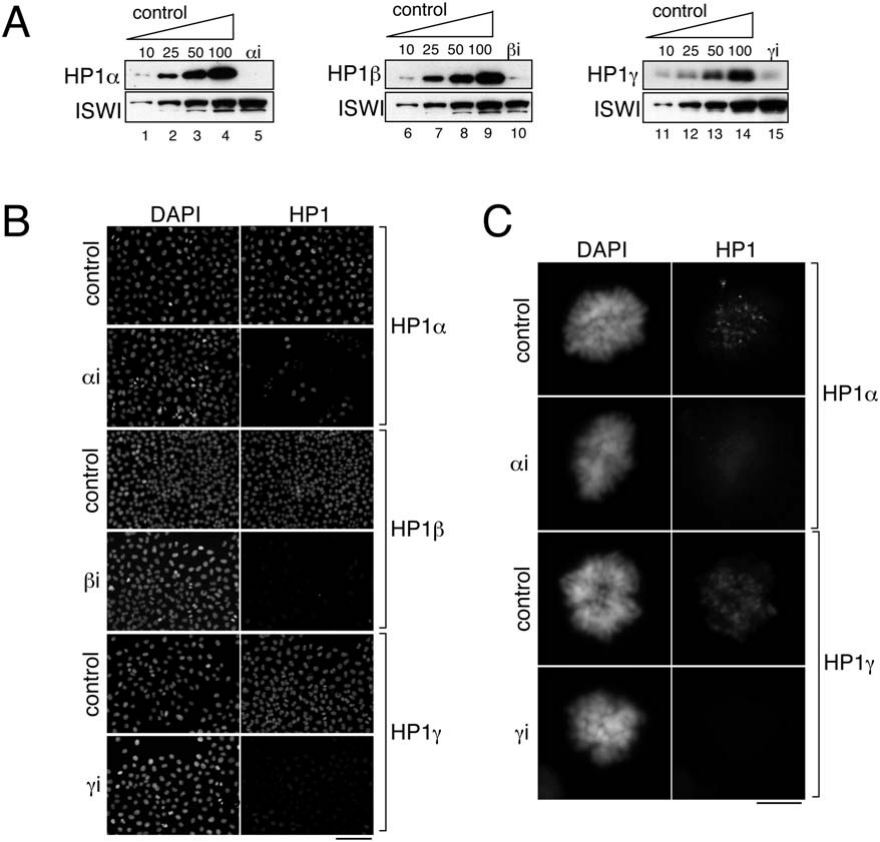
Knock down of HP1 proteins by siRNA in HeLa cells. (A) Extracts made from HeLa cells transfected with siRNAs specific to HP1alpha (lane 5), HP1beta (lane 10) or HP1gamma (lane 15) were analyzed by immunoblotting. To estimate the extent of the depletion of the corresponding isoform, increasing amounts of a control cell extract were loaded on the same gel (lanes 1–4, 5–9 and 11–14). The levels of the chromatin remodeller ISWI were analyzed as a loading control. (B&C) HeLa cells transfected as in A were analyzed by immunofluorescence with the indicated antibodies (green) and counterstained with DAPI (blue). In B, cells were not pre-extracted before fixation. In C, cells were pre-extracted before fixation to detect only the chromatin-bound population. Representative examples of mitotic cells are shown. Scale bars: 50 micrometers in B and 5 micrometers in C.

siRNA oligonucleotides directed against each of the three HP1 isoforms were introduced separately in HeLa cells. Quantitative immunoblotting performed 120 hours after transfection showed that, in all cases, the siRNA treatment reduced the cellular levels of the corresponding isoform around 90% (Figure 3A). This reduction was confirmed by immunofluorescence (Figure 3B). Importantly, disappearance of the chromatin-bound population of HP1 proteins was observed also in mitotic cells (Figure 3C and Figure S2). This is particularly significant since it is at this region that cohesin accumulates in mitosis.

### HP1 depletion does not affect cohesin loading

**Figure 4:**
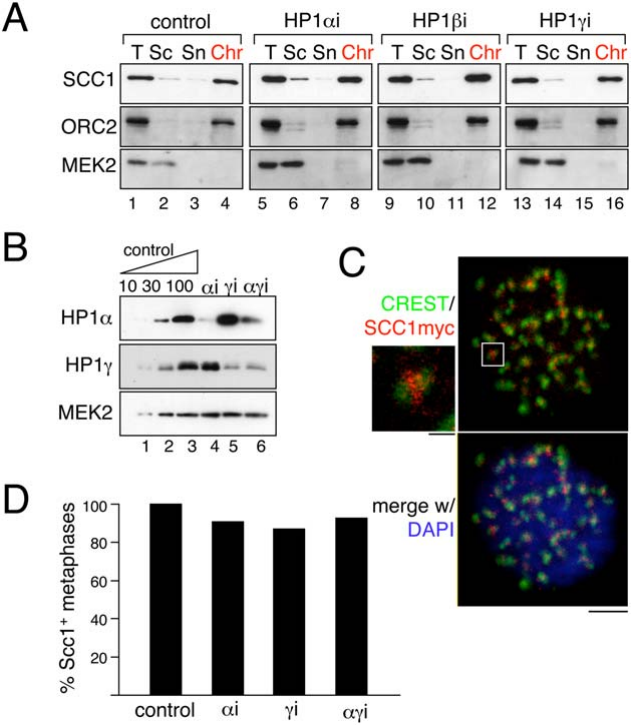
HP1 knock down cells show no defect in cohesin recruitment. (A) HeLa cells transfected with no siRNA (control, lanes 1–4) or siRNAs specific to HP1alpha (lanes 5–8), HP1beta (lanes 9–12) or HP1gamma (lanes 13–16), were separated in three fractions: a soluble cytoplasmic fraction (Sc), a soluble nucleoplasmic fraction (Sn) and a chromatin-enriched fraction (Chr). T is total cell extract. ORC2 and MEK2 are chromatin-bound and cytoplasmic proteins, respectively, that serve as control for the fractionation protocol. (B) A HeLa cell line expressing Scc1-9xmyc under the control of doxicycline was transfected with siRNAs against HP1alpha, HP1gamma, or both, and the remaining levels of each protein were assayed by quantitative immunoblotting five days after transfection. (C) The same cells were grown on coverslips, pre-extracted before fixation and stained with anti-myc (red) and CREST serum (green) and counterstained with DAPI (blue). As expected, the Scc1-myc signal appears between the two sisters centromeres labeled by CREST (inset). Scale bars, 5 micrometers and 1 micrometer (inset on the left). (D) Fraction of mitotic cells showing the staining depicted in (C) relative to control cells (see materials and methods).

Cohesin is loaded on chromatin in early G1 in human cells and most of it remains bound until prophase. To test whether knock down of HP1 proteins has any effect on the recruitment of cohesin to chromatin, we examined the amount of chromatin-bound cohesin after siRNA transfection. The cytoplasmic kinase MEK2 and the chromatin-enriched subunit of the origin recognition complex ORC2 were used as controls for the cell fractionation protocol [46]. As expected, most cohesin is detected in the chromatin fraction and only a small amount is present in the soluble fraction, either cytoplasmic or nucleoplasmic (Figure 4A, lanes 1–4). No noticeable decrease in the levels of chromatin-bound cohesin was observed in cells with reduced levels of HP1alpha, HP1beta or HP1gamma (Figure 4A).

We reasoned that if HP1 knock down affects only the recruitment of cohesin to pericentric heterochromatin (which accounts for less than 15% of the human genome), then a defect in cohesin loading might not be detected with the protocol just described. Thus, we decided to look specifically at the pericentric population of cohesin by immunofluorescence after siRNA transfection. We depleted HP1alpha, HP1gamma or both, because they are specifically located at the centromeric region of mitotic chromosomes (Figure 4B). Detection of the centromeric population of cohesin is technically challenging and antibodies against cohesin subunits do not usually detect it. Instead, a HeLa cell line expressing myc-tagged Scc1 is commonly used for detection of Scc1 with a myc antibody [12]. Mitotic cells were collected after HP1alpha and HP1gamma-siRNA treatment of this cell line, pre-extracted with detergent before fixation and analyzed by staining with anti-myc and a CREST serum that labels the centromeres (Figure 4C). The number of cells showing cohesin staining between sister centromeres was similar in control cells and cells lacking HP1alpha or gamma, or both HP1 isoforms (Figure 4D). Thus, depletion of HP1 proteins does not seem to affect the recruitment of cohesin to chromatin, either to arms or centromeres, in human cells.

### TSA and AZA treatments have no effect on cohesin targeting

**Figure 5:**
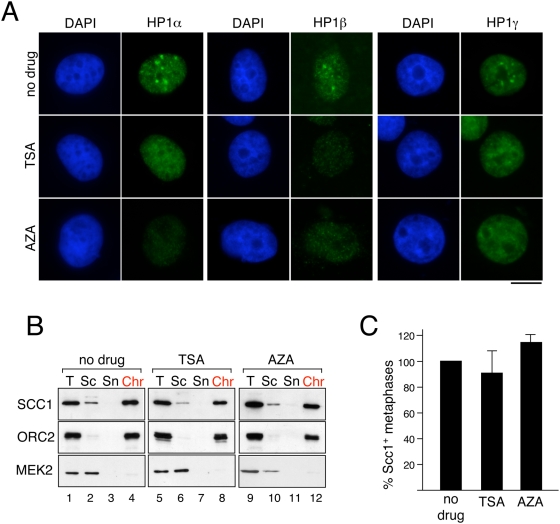
TSA and AZA treatments do not affect cohesin recruitment. (A) Hela cells grown for five days in the presence of 30 ng/mL of TSA, 1 micromolar AZA or no drug, were pre-extracted before fixation, fixed and stained with DAPI (blue) and antibodies against the three HP1 isoforms (green). Scale bar, 10 micrometers. (B) Immunoblot analysis of the chromatin fractions obtained from untreated and TSA- and AZA-treated cells, as described in Figure 4A. (C) Quantitation of Scc1-myc positive mitotic cells, as described in Figure 4D.

As an alternative approach to the study of the influence of heterochromatin in cohesin recruitment, we used drugs that alter the histone modification or the DNA methylation profile of chromatin. HeLa cells were exposed to low doses of the histone deacetylase inhibitor trychostatin A (TSA) or the DNA methylation inhibitor 5-azacytidine (AZA). The effectiveness of the TSA treatment was checked by immunofluorescence. After five days on TSA, cells showed a clear increase in histone H4 acetylation at lysine 8 (Figure S3A). In addition, we observed relocalization of the centromeres to the nuclear periphery, as reported before (e.g., [47], Figure S3B). In the case of cells exposed to AZA, demethylation of the CpG islands present in the pericentromeric satellite 2 was confirmed by bisulfite treatment (Figure S3C). In both TSA-treated and AZA-treated HeLa cells, HP1 proteins were no longer concentrated in heterochromatic foci, but evenly distributed throughout the nucleoplasm, and in some cases their cellular levels appeared to be reduced (Figure 5A).

Chromatin fractionation of control and drug-treated cells showed no evidence for an effect of the treatment in bulk cohesin recruitment (Figure 5B). When the presence of pericentric cohesin was scored in the myc-Scc1 HeLa cell line by immunofluorescence analysis, we did not see a significant reduction in the number of Scc1-myc-positive mitotic cells upon drug treatment (Figure 5C). Thus, delocalization of HP1 proteins following alteration of the histone acetylation and DNA methylation profiles of heterochromatin does not affect cohesin loading and accumulation at centromeres.

### Mitotic defects in HP1gamma knock down cells

**Figure 6:**
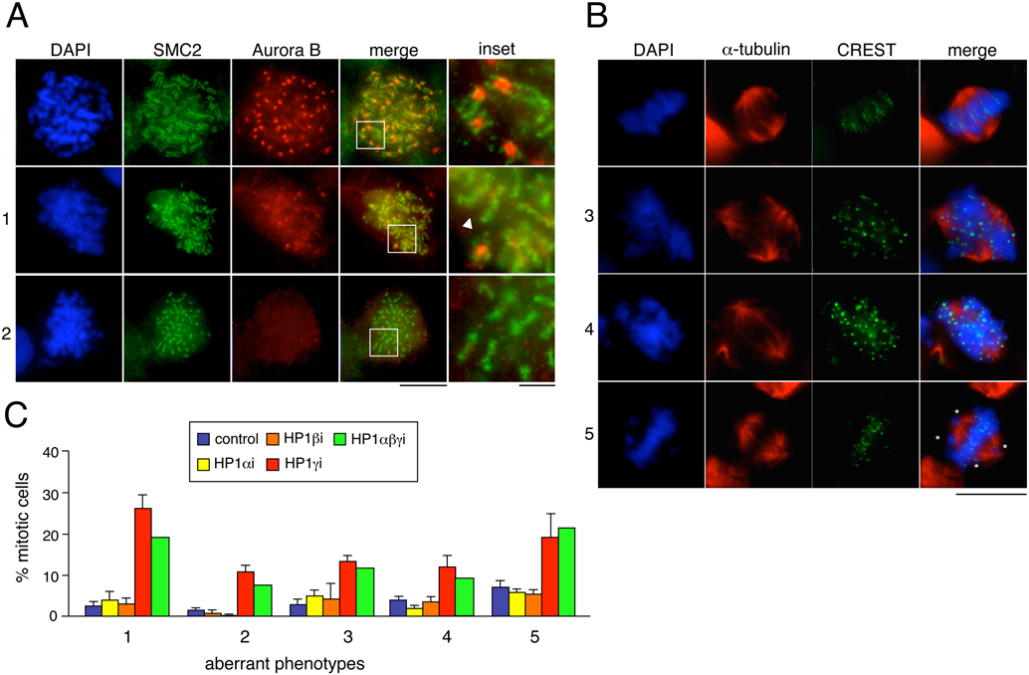
Mitotic defects after HP1gamma reduction in human cells. (A) HeLa cells transfected with siRNAs against the HP1 isoforms were grown on coverslips were incubated for 2 hours in colcemid and then in 60 mM KCl for 30 min, fixed and stained with DAPI (blue), anti- SMC2 condensin subunit (green) and anti-Aurora B (red). Representative examples of a normal metaphase cell (top row), and of the two types of abnormalities scored, labeled “1” and “2” (see text for details). Scale bar, 10 micrometers and 2 micrometers for the inset. The white arrowhead points to a chromosome that has lost the centromeric localization of Aurora B. (B) Cells treated as in A were fixed and stained with DAPI (blue) anti-alpha tubulin (red) and CREST serum (green). In a typical metaphase cell, chromosomes are at the spindle equator, forming a tight metaphase plate (top row). Deviations form this phenotype are: multipolar spindles (“3”, second row), pseudoanaphase (“4”, third row) or bipolar spindles in which a number of chromosomes (from one to five, asterisks) had not yet congressed to the metaphase plate (“5”, bottom row). Scale bar, 10 micrometers. (C) Quantitation of the different phenotypes among the mitotic cell population of control and HP1 knock down cells. More than 200 mitotic cells were scored in two or more independent experiments.

Although our results did not evidence reduced levels of cohesin on chromosomes upon knock down or delocalization of HP1 proteins, it was still possible that cohesin function was altered under these conditions. To test this possibility, we examined the morphology of mitotic chromosomes in cells in which the three HP1 isoforms had been depleted individually or in combination. After a short treatment with colcemid to prevent anaphase, cells were subject to a hypotonic treatment, fixed, and incubated with antibodies against the condensin subunit SMC2 that labels the axis of each sister chromatid and against Aurora B. Most mitotic cells in the control population exhibited chromosomes with well paired sister chromatids along the their entire length, and Aurora B localized to a single dot at the primary constriction (Figure 6A, top row). In some cells, chromosomes had less tightly paired sister chromatids and delocalization of Aurora B from centromeres in a number of chromosomes (labeled as phenotype “1”, Figure 6A, second row). Another phenotype was characterized by overcondensed chromosomes in which the distance between the sister chromatids, both at arms and at centromeres, was clearly increased with respect to control chromosomes and the centromeric Aurora B signal was lost (labeled as “2” in Figure 6A, third row). This phenotype is the most frequently found upon knock down of cohesin subunits (e.g., [48]). Quantitation of the occurrence of these phenotypes among the control and HP1 depleted cells indicates that only in the case of HP1gamma knock down there is a significant increase in phenotype “1” as well as a small increase in phenotype “2” (Figure 6C).

We also scored the presence of aberrant mitotic figures among the control and siRNA-treated cells. For that we omitted the colcemid treatment, fixed the cells and labeled them with antibodies against alpha-tubulin and CREST serum. In a control metaphase, chromosomes become bi-oriented at the spindle equator, and form a tight metaphase plate (Figure 6B, top row). In some cases, however, we observed multipolar spindles (phenotype “3”), pseudoanaphase with scattered chromosomes along the elongated spindle (phenotype “4”), or cells with a bipolar spindle in which a number of chromosomes (from one to five) had not congressed yet to the metaphase plate (phenotype “5”, Figure 6B). Again, quantitation of these mitotic figures phenotypes revealed no defects after HP1alpha or HP1beta knock down, whereas cells with reduced levels of HP1gamma showed at least a 3-fold increase in the occurrence of these aberrant mitotic figures with respect to control cells (Figure 6C). Importantly, co-depletion of the three HP1 isoforms did not increase the defects (Figure 6C and Figure S4), suggesting that they are likely caused by depletion of HP1gamma alone. Thus, our results indicate that the HP1gamma isoform has a more prominent role than the other two in mitotic progression. Given the data presented in previous sections, however, this role appears not to be exerted directly on cohesin recruitment.

## Discussion

Since it was reported that HP1/Swi6 is required for proper centromeric cohesion in *S. pombe*, it has been widely assumed that this mechanism is evolutionary conserved and functions also in higher eukaryotes (e.g., [49]). However, the results presented here and those from other recent publications discussed below support a different conclusion. To date, no physical interaction between cohesin and HP1 proteins has been observed in metazoan organisms ([42], [45], [50], this study). We have found that depletion of HP1 isoforms by siRNA does not perturb bulk cohesin loading on chromatin in interphase nor the presence of cohesin in the pericentric regions of metaphase chromosomes. Importantly, similar results were observed upon treatment of cells with TSA and AZA that prevent the accumulation of HP1 proteins at heterochromatin foci.

It has been recently reported that HP1 proteins interact with hSgo1, the protector of centromeric cohesin, and that the presence of HP1alpha is important to maintain hSgo1 at centromeres [45]. In contrast, we have not detected a reduction of hSgo1 signals in the mitotic chromosomes from HP1-depleted cells (data not shown). Of note, Yamagishi and colleagues show that hSgo1 recruitment to centromeres in prophase is not altered in the absence of HP1, but only its maintenance after prolonged arrest with nocodazole. Differences in the drug treatment could thus explain the discrepancy with our results. Importantly, however, both studies coincide to point out that HP1 proteins have no role in cohesin recruitment to centromeres in human cells.

Double depletion of HP1alpha and HP1gamma from human cells results in micronuclei formation, an indication of aberrant chromosome segregation in mitosis [51]. In our study, single depletion of HP1gamma was sufficient to cause mitotic defects (Figure 6). We suggest that HP1gamma is more important than HP1alpha or HP1beta to ensure a correct progression through mitosis, at least in transformed human cells. This specificity may be variable depending on the species, the tissue, or even the developmental stage [52].

According to our results, the mitotic function of HP1 is not related to cohesin recruitment. Instead, HP1gamma could be implicated in proper kinetochore function through interaction with Mis12 [51], or it could be required for proper accumulation of Aurora B at centromeres (Figure 6, [53], [54]). In any of these cases, defects in mitosis could result in loosened cohesion due to prolonged metaphase arrest. In addition, early studies of Drosophila mutant embryos proposed that the requirement of HP1 for proper chromosome segregation could be attributed to a role in chromosome condensation [55]. More recently, the participation of HP1 in chromosome condensation in human cells has also been suggested [56]. Consistent with this report, the morphology of the metaphase chromosomes labeled as “1” in Figure 6, which represent up to 30% of the mitotic cells upon HP1gamma depletion, resemble those described in condensin II depleted cells [57]. The functional significance of this resemblance awaits further investigation. Given the role of HP1 proteins as transcriptional regulators, it is also possible that the defects observed upon its depletion are the indirect consequence of altered expression of proteins involved in chromosome segregation [58], [59].

In fission yeast and Drosophila, RNA interference plays a fundamental role in the establishment and maintenance of heterochromatin [60]. Whether this is also true for mammalian cells remains to be proven. A conditional loss-of-function mutant of the dsRNA cleaving enzyme Dicer in a chicken-human hybrid cell line shows mitotic defects concomitant with delocalization of HP1 and cohesin from centromeres [61]. The authors of this study suggest that HP1 is responsible for the recruitment of cohesin and checkpoint proteins and that this pathway requires Dicer function. Consistent with this hypothesis, bivalent chromosomes of mouse oocytes deficient for Dicer show decreased cohesion [62]. In contrast, cohesion defects are not apparent in Dicer^−/−^ mouse ES cells despite the severe reduction in H3K9Me and delocalization of HP1 [63].

In mouse embryonic fibroblasts lacking the Suv39h histone methyltransferases there is no accumulation of HP1 proteins in heterochromatin. However, cohesin remains associated with the major satellite in the pericentric region [42]. Interestingly, the chromosomes of Suv39h^−/−^ cells show decreased cohesion in the region of the major satellite DNA, but not in the more centromeric region of the minor satellite, where kinetochores are assembled and where, presumably, cohesin is protected from the prophase dissociation pathway by Shugoshin [41]. Thus, the cohesion observed at the major satellite region of metaphase chromosomes in wild type MEFs (and in the pericentric heterochromatin regions of other organisms) may not rely on cohesin. DNA catenation resulting from the replication process also contributes to cohesion [64]–[66]. One possibility is that the compaction of heterochromatin hinders the action of topoisomerase II on DNA catenations. In the Suv39h^−/−^ mouse cells, the altered structure of heterochromatin might facilitate the decatenation process in the major satellite region and lead to arm separation once cohesin has been removed from this region by the prophase pathway. Clearly, further studies are required to understand the molecular mechanisms underlying the link between heterochromatin and sister chromatid cohesion.

## Materials and Methods

### Antibodies

A list of the antibodies used in this study appears in the Supporting Information (Text S1).

### Immunofluorescence

HeLa cells grown on coverslips were fixed with 2% paraformaldehyde (PFA) in PBS (pH 7.4) for 15 min and permeabilized in 0.2% TritonX-100 in PBS for 4 min at room temperature. For hypotonic treatment, cells were incubated in 60 mM KCl at room temperature for 30 min before fixation. When required, cells were pre-extracted with 0.5% Triton X-100 in CSK buffer (10 mM Pipes pH 7.0, 100 mM NaCl, 3 mM MgCl_2_ and 300 mM sucrose) for 5 min before fixation. For the analysis of mitotic cohesin in the Scc1-myc HeLa cell line (a kind gift of J.M. Peters, [11]), cells were arrested in mitosis in 0.1 microgram/ml of colcemid for 20 hour before analysis, and they were then pre-extracted with 0.1% Triton X-100 in PBS for 4 min at room temperature. Since not every cell in the culture expresses the Scc1-myc protein, the relative percentage of Scc1^+^ metaphases was calculated with the formula *100 x I_c_ x M_t_ / M_c_ x I_t_* where *I* is the fraction of myc-positive interphase cells, *M* is the fraction of metaphase cells showing a myc signal between the two centromere dots labeled by CREST (as in Figure 4C), and the subindexes *c* and *t* denote control and treated cells, respectively.

Metaphase spreads prepared by cytospin were immersed in KCM (120 mM KCl, 20 mM NaCl, 10 mM Tris-HCl pH 7.7, 0.5 mM EDTA and 0,1% TritonX-100), blocked in 3% BSA in KCM for 30 min and incubated with primary and secondary antibodies in the same buffer for 1 hr. Cells were fixed in 2% PFA in KCM for 10 min before staining with 1 microgram/ml DAPI. A Leica DM6000 microscope was used to obtain grayscale images, which were later pseudo-colored and merged using Adobe Photoshop. For the images shown in Figure 1, 4C and S3B, a confocal microscope Leica TCS-SP5 (AOBS) was used.

### Immunoprecipitation

Cell extracts used for immunoprecipitation of endogenous cohesin and HP1 were prepared by resuspending HeLa cells in osmotic buffer A (200 mM KCl, 40 mM Tris pH 7.5, 0.34 M sucrose, 10% glycerol, 1 mM DTT, 1 mM NaVO_4_, 5 mM beta-glycerophosphate, 0.1 mM PMSF, 5 mM NaF and 1× protease inhibitor cocktail from Roche) at 3×10^7^ cells per ml and disrupting them with a dounce grinder. Pelleted cells were resuspended in hypotonic buffer (buffer A without glycerol and sucrose) and digested with micrococcal nuclease (0.2 units/ml) at 25°C for 25 minutes and placed on a rotating wheel at 4°C for 15 minutes. After adding 2 mM EGTA the soluble extract was recovered by centrifugation at 16,000×g at 4°C. For each immunoprecipitation reaction 0.25 mg of protein and 2 micrograms of antibody were used. Immunoprecipitation of HP1 after cross-linking was performed as described [45].

Recombinant mouse HP1alpha, HP1beta, HP1gamma, cloned in pGEX2TK (obtained from P. Chambon), were purified as GST fusions from *E. coli*. Twenty micrograms of each protein (or of GST alone) were bound to 10 microliters of GST agarose beads and incubated with 100 microliters of HeLa cell nuclear extract. After extensive washing, the proteins bound to the beads were analyzed by immunoblotting.

### siRNA

Exponentially growing HeLa cells were transfected twice with 100 nM oligo RNA duplexes using Oligofectamine (Invitrogen) at 0 and 24 hr. Control cells were transfected with a mixture containing no siRNA. Cells were seeded on wells containing poly-lysine-coated coverslips at 48 hr and processed for immunofluorescence at 120 hr. Total cell extracts were also prepared at this time to check the extent of depletion by immunoblotting. The sequences of the sense strand of the siRNA duplexes (Stealth siRNA, Invitrogen) used were: 5′UAACAAGAGGAAAUCCAAUUUCUCA3′ (HP1alpha), 5′GGAUAAGUGUUUCAAGGCAACCUUU3′ (HP1beta), 5′UCUUAACUCUCAGAAAGCUGGCAAA 3′ (HP1gamma).

### Chromatin fractionation

For chromatin fractionation, we used the protocol of Méndez and Stillman [46]. In brief, cells were resuspended (1×10^7^ cells/ml) in buffer containing 10 mM HEPES pH 7.9, 10 mM KCl, 1.5 mM MgCl_2_, 0.34 M sucrose, 10% glycerol, 1 mM DTT, and 1× protease inhibitor cocktail (Roche). Triton X-100 (0.1%) was added, and the cells were incubated for 5 min on ice. Nuclei were collected by low-speed centrifugation (4 min, 1300×g, 4°C) and the supernatant was further clarified by high-speed centrifugation (15 min, 20,000×g, 4°C). Nuclei were lysed in a buffer B containing 3 mM EDTA, 0.2 mM EGTA, 1 mM DTT and protease inhibitors. Insoluble chromatin was separated from soluble nuclear proteins by centrifugation (4 min, 1,700×g, 4°C). The final chromatin pellet was resuspended in Laemmli buffer and sonicated before being boiled and analyzed by SDS-PAGE and immunobloting.

### TSA and AZA treatments

Exponentially growing Hela cells were cultured for five days in medium containing 30 ng/ml TSA (Sigma) or 1 micromolar 5-azacytidine (Sigma), with daily changes of media. Cells were split over coverslips on the third day and taken for analysis on the fifth day.

## Supporting Information

Text S1 (0.05 MB DOC)

Figure S1 Localization of HP1 isoforms in HeLa cells in interphase and mitosis. Exponentially growing HeLa cells grown on coverslips were fixed without (−) or with (+) pre-extraction and stained with antibodies against the different HP1 isoforms (green) and DAPI (blue). Representative examples of interphase and mitotic cells are shown. Scale bar, 10 micrometers.
(4.38 MB TIF)

Figure S2 HP1alpha and HP1gamma are not localized at the centromere upon siRNA knock down. Representative images of metaphase spreads from HeLa cells transfected with siRNAs against HP1alpha and HP1gamma stained with the indicated HP1 antibody (red), CREST serum (green) and DAPI (blue). Scale bars, 10 micrometers and 5 micrometers (inset).
(5.06 MB TIF)

Figure S3 Efficiency of the TSA and AZA treatments. (A) Hela cells grown for five days in the absence or presence of 30 ng/mL TSA were fixed and stained with DAPI and an antibody that recognizes histone H4 acetylated on Lysine 8 (H4AcK8). The increased staining of TSA-treated cells confirms the effectiveness of the treatment. Bar, 100 micrometers. (B) Confocal sections of a control cell and a cell treated with TSA and stained with DAPI and CREST serum (blue and green, respectively, in the merged image). The TSA treatment induces relocalization of centromeres to the nuclear periphery. Scale bars, 10 micrometers. (C) Methylation of the CpG dinucleotides of pericentromeric satellite 2 (sat2) in cells untreated and treated with 5 micromolar AZA for five days was checked by bisulfite treatment followed by PCR and sequencing analysis. Each square represents a methylated (black) or unmethylated (white) CpG at the indicated positions within the sat2 sequence.
(4.01 MB TIF)

Figure S4 Triple depletion of HP1 isoforms by siRNA. An extract made from HeLa cells transfected with a combination of HP1alpha-, HP1beta-, and HP1gamma-siRNA was analyzed by immunoblotting. To estimate the extent of the depletion of the each isoform, increasing amounts of a control cell extract were loaded on the same gel (lanes 1–3). The levels of MEK2 were analyzed as a loading control.
(0.21 MB TIF)
