# Supplementary material for: Heterochromatin Protein 1 (HP1) Proteins Do Not Drive Pericentromeric Cohesin Enrichment in Human Cells
Source: PLoS One. 2009 Apr 8;4(4):e5118. doi: 10.1371/journal.pone.0005118 (PMC2662427; doi:10.1371/journal.pone.0005118)
Supplement: Text S1 — (0.05 MB DOC) [file pone.0005118.s001.doc]

**Supporting Information**

**Materials and Methods**

*Antibodies*

The following antibodies were used in this study: HP1alpha (clone 15.19s2, Upstate), HP1beta (1MOD 1A9, Eurogentec), HP1gamma (clone MAB3450, Chemicon), Aurora B (AIM-1) and MEK2 (BD Biosciences), alpha-tubulin (clone DM1A, Sigma), myc (clone 9E/10), ORC2 (obtained from J. Méndez [1]), CREST serum (obtained from Y. Muro [2]), hSMC2 [3], SMC3 [4], ISWI [5], acetyl-histone H4 (Upstate). A polyclonal antibody against hSgo1 was generated by immunizing rabbits with a His-tagged recombinant protein corresponding to aminoacids 30 to 307 (Innovagen).

*Bisulfite treatment*

After treatment with AZA, the DNA methylation status of the CpG dinucleotides present in pericentromeric satellite 2 (sat2) repeats was established by bisulfite treatment of DNA, which induces chemical conversion of cytosine residues to uracil, but leaves 5-methylcytosine residues unaffected. Sat2 repeats were amplified by PCR with the primers 5´-ATGGAAATGAAAGGGGTTATTATT-3´ and 5´- AAATTATTCCATTCCATTCCATTAA-3´, cloned in pGEM-T-EASY vector (Promega) and sequenced.

**References**

1. Mendez J, Stillman B (2000) Chromatin association of human origin recognition complex, cdc6, and minichromosome maintenance proteins during the cell cycle: assembly of prereplication complexes in late mitosis. Mol Cell Biol 20: 8602-8612.

2. Muro Y, Matsumoto Y, Ohashi M (1992) Anticentromere-protein-B--DNA complex activities in anticentromere antibody-positive patients. Arch Dermatol Res 284: 396-399.

3. Kimura K, Cuvier O, Hirano T (2001) Chromosome condensation by a human condensin complex in Xenopus egg extracts. J Biol Chem 276: 5417-5420.

4. Losada A, Hirano M, Hirano T (1998) Identification of Xenopus SMC protein complexes required for sister chromatid cohesion. Genes Dev 12: 1986-1997.

5. MacCallum DE, Losada A, Kobayashi R, Hirano T (2002) ISWI remodeling complexes in Xenopus egg extracts: identification as major chromosomal components that are regulated by INCENP-aurora B. Mol Biol Cell 13: 25-39.
