# Supplementary figures and images for: Heterochromatin Protein 1 (HP1) Proteins Do Not Drive Pericentromeric Cohesin Enrichment in Human Cells
Source: PLoS One. 2009 Apr 8;4(4):e5118. doi: 10.1371/journal.pone.0005118 (PMC2662427; doi:10.1371/journal.pone.0005118)

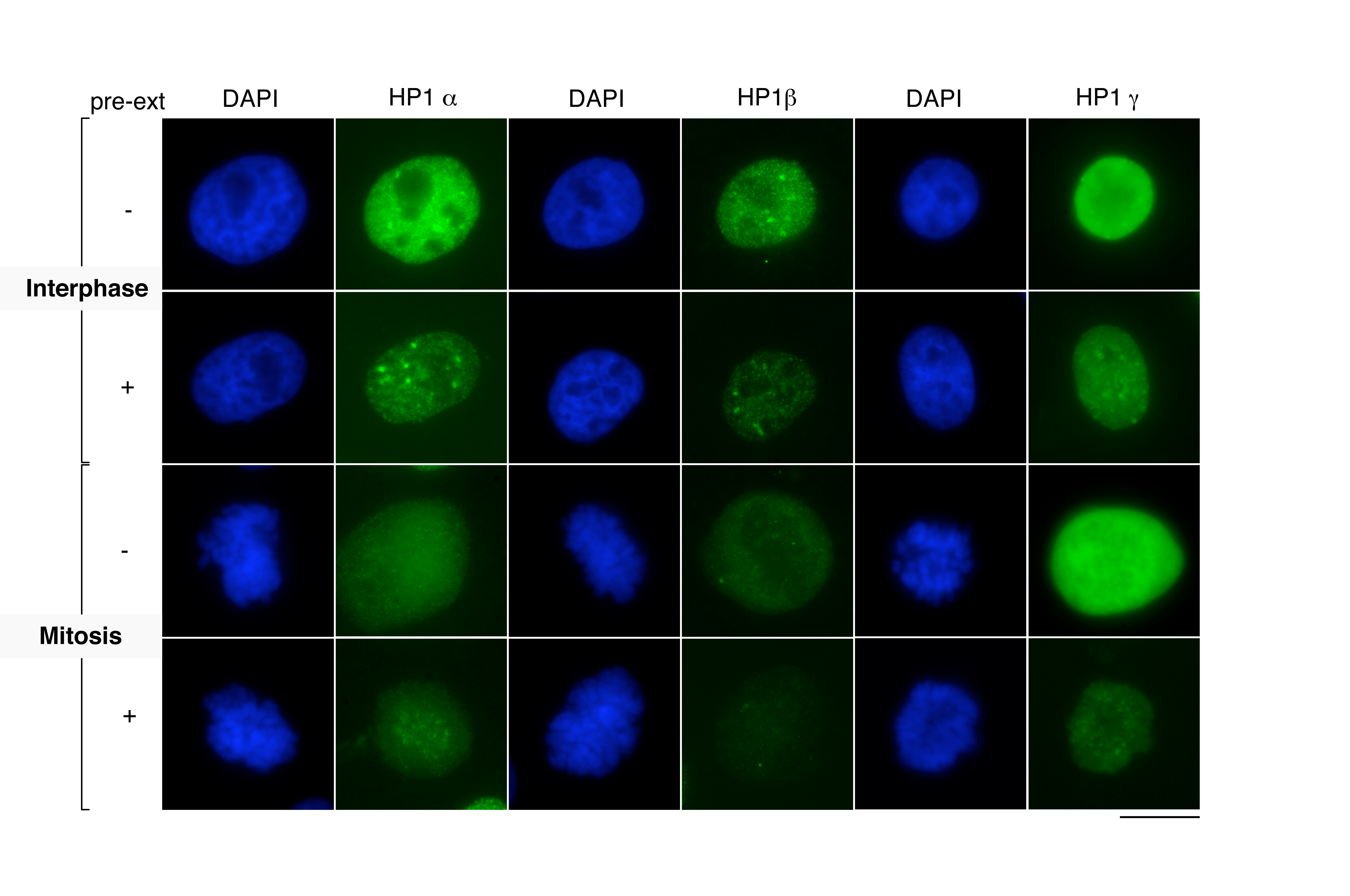

Supplement: Figure S1 — Localization of HP1 isoforms in HeLa cells in interphase and mitosis. Exponentially growing HeLa cells grown on coverslips were fixed without (−) or with (+) pre-extraction and stained with antibodies against the different HP1 isoforms (green) and DAPI (blue). Representative examples of interphase and mitotic cells are shown. Scale bar, 10 micrometers. (4.38 MB TIF) [file pone.0005118.s002.tif]

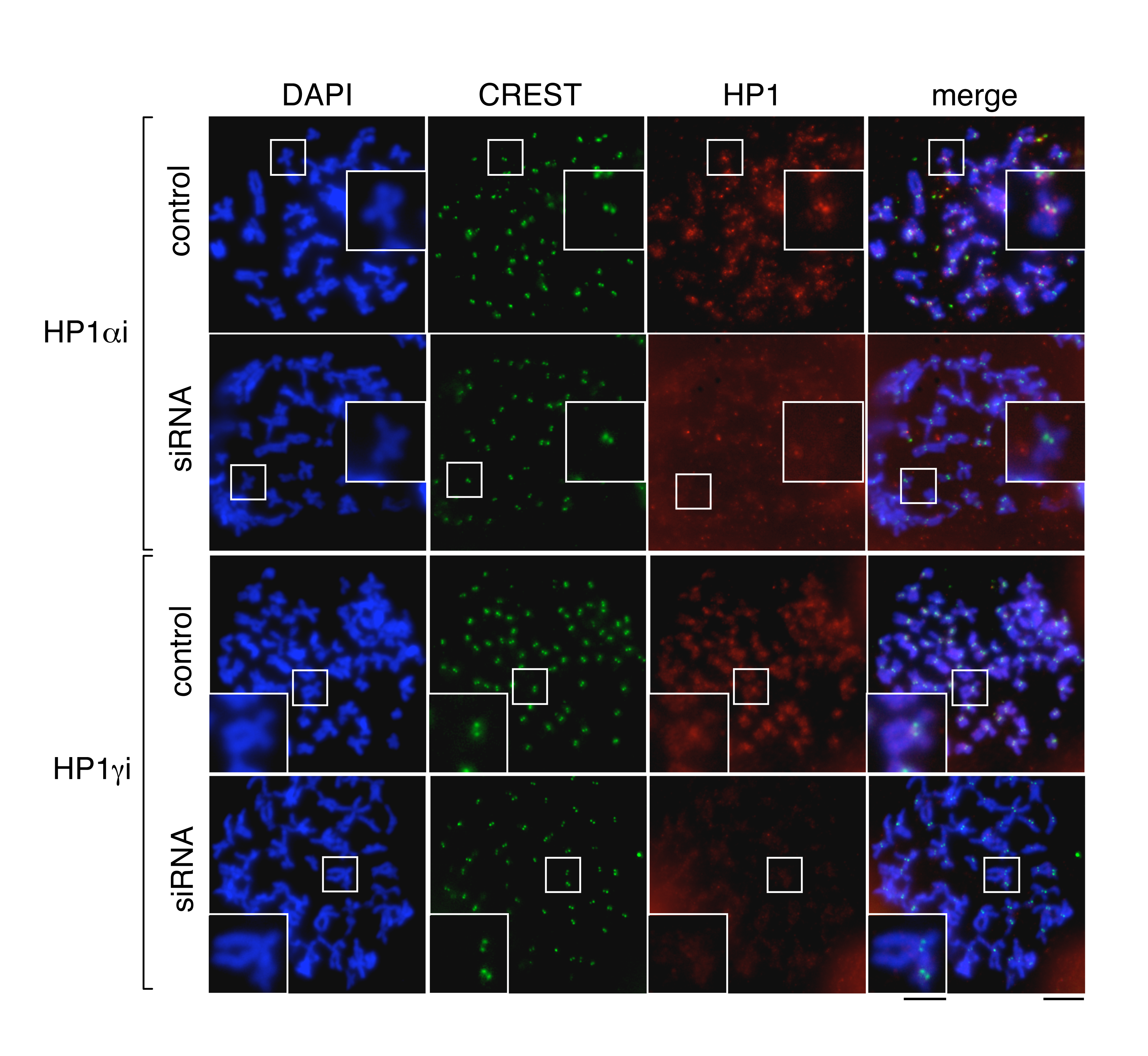

Supplement: Figure S2 — HP1alpha and HP1gamma are not localized at the centromere upon siRNA knock down. Representative images of metaphase spreads from HeLa cells transfected with siRNAs against HP1alpha and HP1gamma stained with the indicated HP1 antibody (red), CREST serum (green) and DAPI (blue). Scale bars, 10 micrometers and 5 micrometers (inset). (5.06 MB TIF) [file pone.0005118.s003.tif]

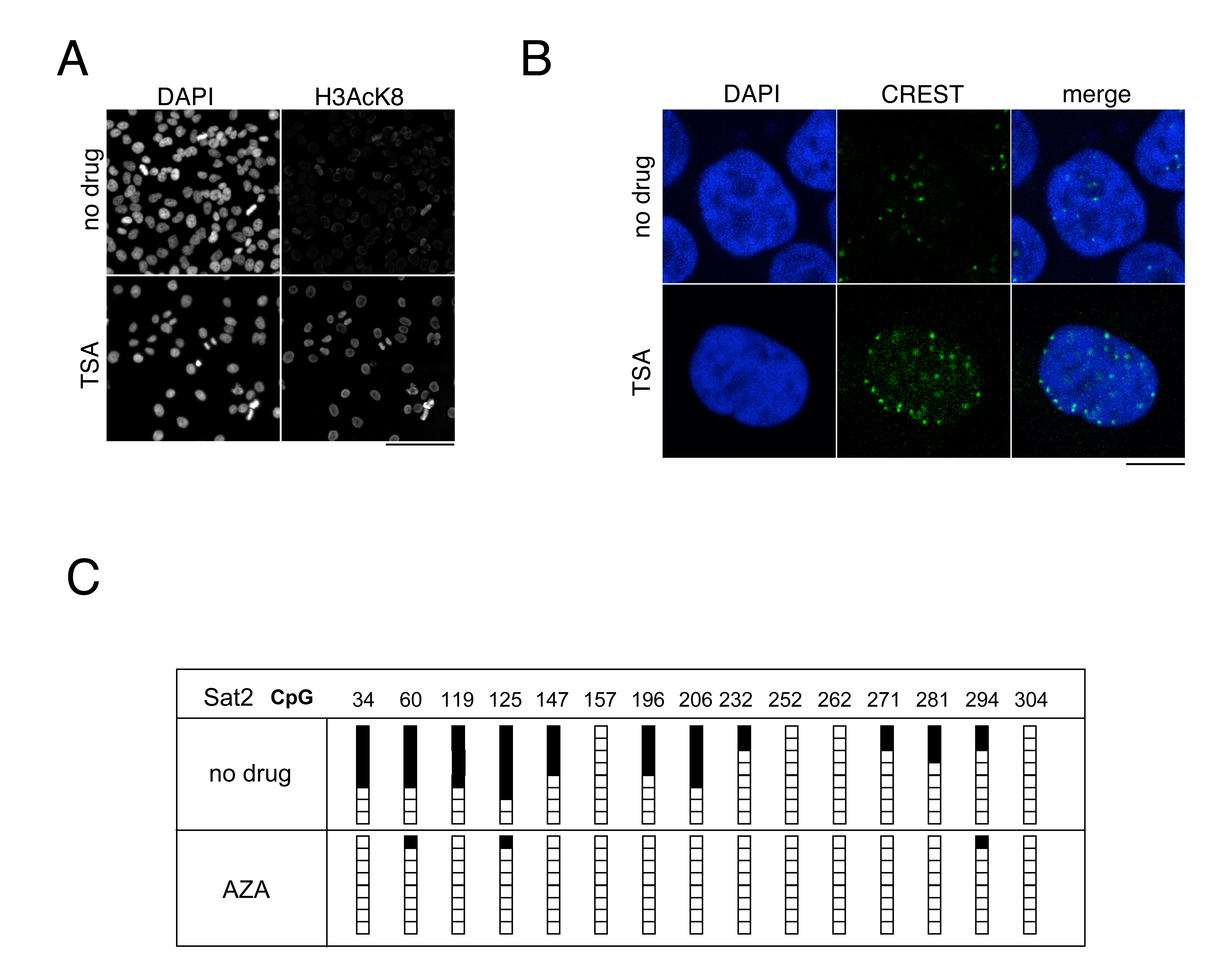

Supplement: Figure S3 — Efficiency of the TSA and AZA treatments. (A) Hela cells grown for five days in the absence or presence of 30 ng/mL TSA were fixed and stained with DAPI and an antibody that recognizes histone H4 acetylated on Lysine 8 (H4AcK8). The increased staining of TSA-treated cells confirms the effectiveness of the treatment. Bar, 100 micrometers. (B) Confocal sections of a control cell and a cell treated with TSA and stained with DAPI and CREST serum (blue and green, respectively, in the merged image). The TSA treatment induces relocalization of centromeres to the nuclear periphery. Scale bars, 10 micrometers. (C) Methylation of the CpG dinucleotides of pericentromeric satellite 2 (sat2) in cells untreated and treated with 5 micromolar AZA for five days was checked by bisulfite treatment followed by PCR and sequencing analysis. Each square represents a methylated (black) or unmethylated (white) CpG at the indicated positions within the sat2 sequence. (4.01 MB TIF) [file pone.0005118.s004.tif]

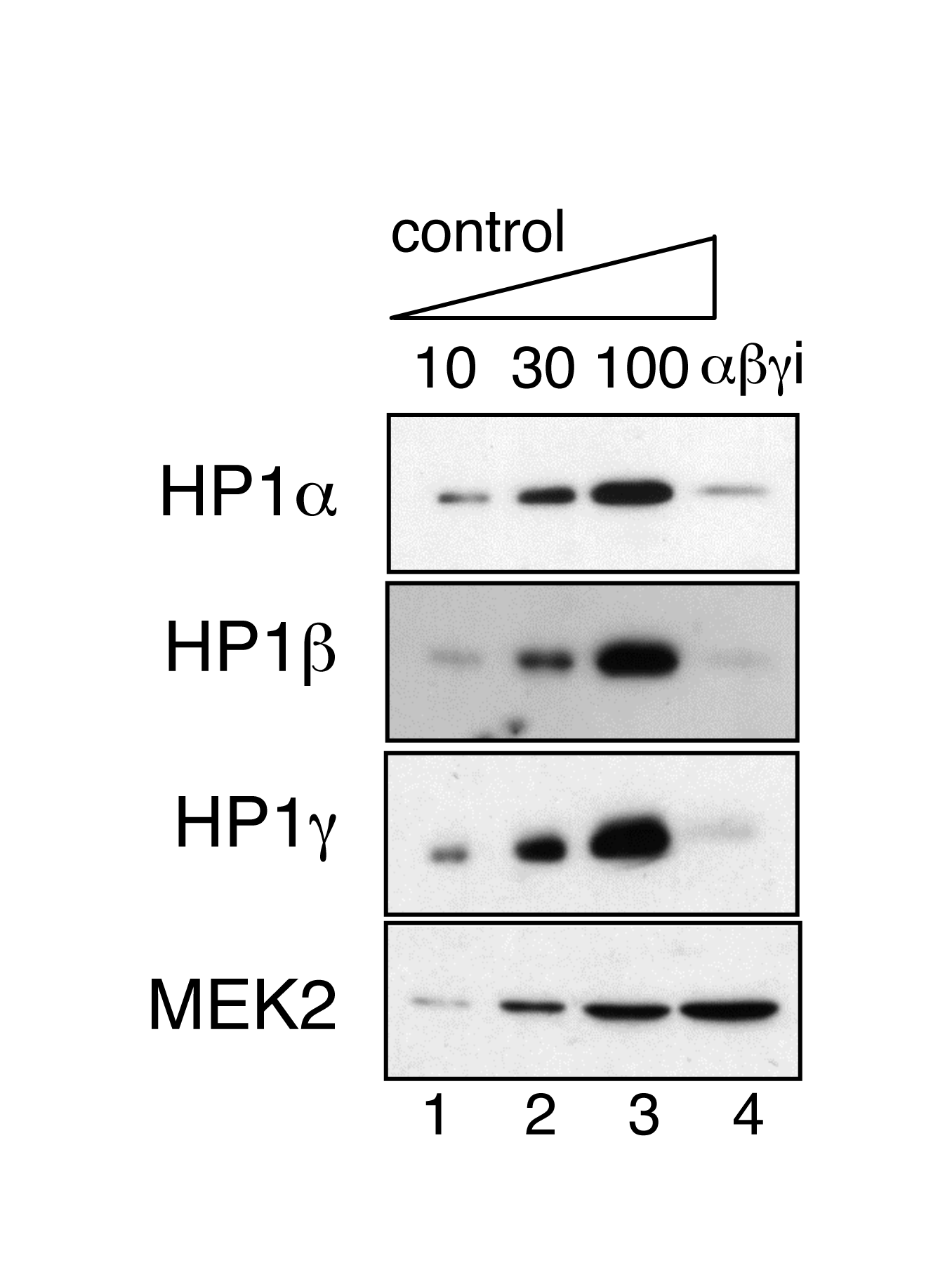

Supplement: Figure S4 — Triple depletion of HP1 isoforms by siRNA. An extract made from HeLa cells transfected with a combination of HP1alpha-, HP1beta-, and HP1gamma-siRNA was analyzed by immunoblotting. To estimate the extent of the depletion of the each isoform, increasing amounts of a control cell extract were loaded on the same gel (lanes 1–3). The levels of MEK2 were analyzed as a loading control. (0.21 MB TIF) [file pone.0005118.s005.tif]
